# Human immunodeficiency virus-related renal cell carcinoma: a retrospective study of 19 cases

**DOI:** 10.1186/s13027-021-00362-7

**Published:** 2021-04-21

**Authors:** Mengmeng Zhang, Zhiqiang Zhu, Wenrui Xue, Hui Liu, Yu Zhang

**Affiliations:** 1 Department of Urology, Beijing Youan Hospital, Capital Medical University, Beijing, China; 2 Department of Pathology, Beijing Youan Hospital, Capital Medical University, Beijing, China

**Keywords:** Human immunodeficiency virus, Renal cell carcinoma, Treatment

## Abstract

**Purpose:**

We aimed to investigate basic information, clinical findings, treatments for tumor, pathology, and outcomes of HIV-positive patients diagnosed with renal cell carcinoma (RCC).

**Patients and methods:**

We collected 19 patients from 2012 to 2020 who are diagnosed with RCC with HIV-positive. A retrospective analysis was performed on their hospitalization course and tumor-related parameters, including basic information, clinical findings, HIV-associated data, pathology, treatments for tumor, and outcomes.

**Results:**

In our study, patients were diagnosed with RCC at the median age of 51. Males took a great part (17 males, 89%) in all patients, while only 2 females were diagnosed. The median CD4^+^ T lymphocyte cell count was 462 cells/μl when diagnosed with RCC (range from 111 cells/μl to 1536 cells/μl). Eleven patients diagnosed with RCC and HIV infection at the same time, who may have high viral load and low CD4^+^ T lymphocyte cell count. Eight patients accepted a median HAART for 30 months (range from 11 months to 108 months) prior to diagnosis of RCC. All the patients performed operations successfully, and 4 of them performed partial nephrecotomy. Only 1 patient was identified with chromophobe cell carcinoma, 1 with partially clear cell and partially papillary carcinoma, and 17 with clear cell carcinoma. Two of the patients with Fuhrman grades 2–3 accepted cytokine therapy with IL-2 and IFN-α. Two patients died of lung metastasis 1 year and 6 months after surgery respectively, even though 1 patient accepted full dose targeted therapy (sorafenib) for 3 months, and one refused adjuvant therapy. The remaining 17 patients are still alive at a median follow-up of 34 months; however, 1 patient lives with lung and brain metastases at the last follow-up of 3 years after surgery.

**Conclusions:**

RCC patients with HIV-positive were similar to the general population in terms of clinical characters, treatment measures, and pathology. RCC patients with HIV-positive seemed like to obey the same clinical practice guideline as in the general population. The outcomes of HIV-positive patients with partial nephrectomy are not inferior to patients with radical nephrectomy. Furthermore, experience in targeted therapy and immunal therapy (PD-1/PD-L1 inhibitors) needs to be learned.

## Introduction

Human immunodeficiency virus (HIV) infects the body’s dendritic cells (DCs) and macrophages and activates CD4^+^ T cells, causing destructions of the immunal system, and leading to a significant increment of incidence of certain cancers. So medical scientists focus on these acquired immune deficiency syndrome (AIDS)-defining cancers (Kaposi’s sarcoma, non-Hodgkin lymphoma [NHL], and invasive cervical carcinoma) [1]. Following widely used and great advances in highly active antiretroviral therapy (HAART), up to date the situation has been well controlled. In addition to this, an increment in the incidence of other non-AIDS-defining cancers along with improved survival was noted [2]. Renal cell carcinoma (RCC) has become one of the most common genitourinary tumors, and it is the sixth and eighth common cancer representing 5% and 3% of all cancers respectively in males and females in the USA in 2019 [3]. Few cases diagnosed with RCC with HIV infection were reported worldwide in recent years. The cases from the USA, Africa, Europe, and India take accounts for the most, although the latest cases were reported in Australia [4]. In 1984, Gardenswartz et al. firstly reported a case of RCC associated with HIV infection. The patient was a 38-year-old African American man who had Kaposi’s sarcoma and CMV infection at the same time. They considered that the onset of RCC was associated with immunal deficiency caused by HIV infection [5]. France surgeons reported 1 renal adenocarcinoma and 1 renal angiosarcoma and introduced their clinical course, treatment measures, and outcomes of the malignancies [6]. Increased prevalence of RCC in local HIV patients was documented in the USA. The report revealed that the prevalence of RCC in HIV-positive patients was 8.5 times greater than non-HIV population, with a mean age of occurrence approximately 15 years younger than the general population. Similar conclusions were found in Negeria and Uganda [7]. In India, 1 RCC patient was screened from 2880 HIV-infected individuals with good prognosis [8]. A transatlantic case report showed no difference on the clinical presentation and behavior of RCC between HIV-positive patients and HIV-negative patients, and it seemed like that chronic immunosuppression played a lesser role than age and exposure to risk factors in this setting [9]. A statewide report from Australia retrospectively reviewed 9 patients with HIV and RCC in a HIV referral center. The report revealed that patients with HIV and RCC should be treated with the same guideline as the general population, taking into account poor prognosis of HIV [4].

However, the cases diagnosed with RCC with HIV infection were not widely reported worldwide nowadays. We presented 19 cases of patients HIV infection who diagnosed with RCC, and preliminarily attempted to learn if there was an obvious difference between HIV-positive and general population diagnosed with RCC on clinical characters, treatment measures, pathology, especially on outcomes obeying the same guideline as the general population.

## Patients and methods

The cases diagnosed with RCC with HIV infection were screened from the patients who were treated at the Department of Urology in Beijing Youan hospital affiliated to Capital Medical University from 2012 to 2020. Hospital records were checked out, in order to collect patients’ basic information, HIV status, and RCC relevant data. Patients’ basic information included demographics, diagnosis, and comorbidity. The data of HIV status included CD4^+^ T lymphocyte cell count, HIV viral load at diagnosis of RCC, treatment duration of HAART. RCC relevant data included clinical presentation, treatment measures, pathology, and survival outcomes. Descriptive statistical analysis was used for the acquired data.

## Results

### Basic information and HIV status

Nineteen patients diagnosed with RCC with HIV infection were identified from our department of urology in all from 2012 to 2020. Males took account for the majority (89.5%,17cases), while the females were rare (10.5%, 2cases). The median age was 51 years old (range from 30 to 72 years). The most common comorbidity was hypertension, which occurred in four patients. One patient was co-infected with HIV and syphilis. Five patients(26.3%) had been smoking for more than 10 years. Fifteen patients (79%) presented with incidental findings on imaging examination, 1 presented with flank pain and hematuria, 1 presented with flank pain, and 2 presented with hematuria. Eleven patients are diagnosed with RCC and HIV infection at the same time. Eight patients accepted a median HAART for 30 months (range from 11 months to 108 months) prior to diagnosis of RCC. Thirteen patients underwent viral load measurements when diagnosed with RCC, 5 of whom had a viral load of target not detected (TND). The median CD4^+^ T lymphocyte cell count was 462 cells/μl when diagnosed with RCC (range from 111 cells/μl to 1536 cells/μl) (Tables 1 and 2).

**Table 1:** Basic information of the cases and HIV related data

Case	Gender	Age	Comorbidity	Presentation	CD4 count (cells/μL)	Viral load (copies/mL)	Regular HAART before surgery	HAART duration (months)
1	Male	59	Hypertension	Incidental	614	NT	No	-
2	Male	61	None	Incidental	288	102130	No	-
3	Male	43	None	Incidental	390	NT	Yes	12
4	Male	58	None	Incidental	1536	386	No	-
5	Male	45	Hypertension	Incidental	628	NT	No	-
6	Male	51	Diabetes	Incidental	611	NT	Yes	12
7	Male	54	Hypertension Diabetes	Incidental	880	TND	Yes	36
8	Male	51	None	Hematuria	190	6495	No	-
9	Male	34	None	Hematuria	462	NT	Yes	24
10	Male	72	Diabetes insipidus	Flank pain	378	< 40	Yes	108
11	Male	54	None	Incidental	535	63,188	No	-
12	Female	51	None	Incidental	111	409,601	No	-
13	Male	53	Hypertension	Incidental	328	TND	No	-
14	Male	30	Meniere's Disease	Incidental	604	TND	Yes	36
15	Male	39	None	Incidental	296	NT	No	-
16	Male	50	None	Incidental	151	73,907	No	-
17	Male	50	None	Incidental	327	4909	No	-
18	Female	54	None	Hematuria Flank pain	665	TND	Yes	11
19	Male	48	Syphilis	Incidental	589	TND	Yes	60

Abbreviations: *NT* not tested, *TND* target not detected

**Table 2:** Pathology, treatment, and outcomes of HIV related RCC

Case	Surgery	TNM stage	Pathology	Adjuvent therapy	Follow-up duration (months)	Outcomes
1	RN	pT1a N0 M0	Clear cell Grades 1–2	NT	96	Alive
2	PN	pT1a N0 M0	Chromophobe cell NR	NT	78	Alive
3	RN	pT1a N0 M0	Clear cell NR	NT	76	Alive
4	RN	pT2a N0 M0	Clear cell Grades 1–2	NT	68	Dead
5	RN	pT2b N0 M0 (cystic renal carcinoma)	Clear cell NR	NT	65	Alive
6	PN	pT1a N0 M0	Clear cell Grade 3	NT	61	Alive
7	RN	pT1a N0 M0	Clear cell Grades 1–2	NT	47	Alive
8	RN	pT3b N0 M0 (inferior vena cava cancerous suppository)	Partially clear cell, partially papillary Grades 2–3	IFN+IL-2	35	Alive
9	RN	pT2a N0 M0	Clear cell Grades 2–3	IFN Sorafenib 4 M Sunitinib 6 M Axitinib 3 M	35	Alive (Lung and brain metastases)
10	RN	pT3a N0 M0 (perirenal adipose sac invaded)	Clear cell Grade 2	Sorafenib 3 M	35	Dead
11	RN	pT1b N0 M0	Clear cell Grade 3	NT	34	Alive
12	RN	pT1a N0 M0	Clear cell Grade 2	NT	34	Alive
13	RN	pT1b N0 M0	Clear cell NR	NT	33	Alive
14	PN	pT1a N0 M0	Clear cell Grade 2	NT	33	Alive
15	RN	pT1b N0 M0	Clear cell Grade 2	NT	32	Alive
16	RN	pT1b N0 M0	Clear cell Grade 3	NT	29	Alive
17	RN	pT1a N0 M0	Clear cell Grade 2	NT	28	Alive
18	RN	pT1b N0 M0	Clear cell Grade 3	NT	20	Alive
19	PN	pT1a N0 M0	Clear cell Grade 1	NT	14	Alive

Abbreviations: *RN* radical nephrectomy, *PN* partial nephrectomy, *NR* not recorded, *IFN* interferon, *IL* interleukin, *NT* no treatment

### Pathology

**Fig. 1:**
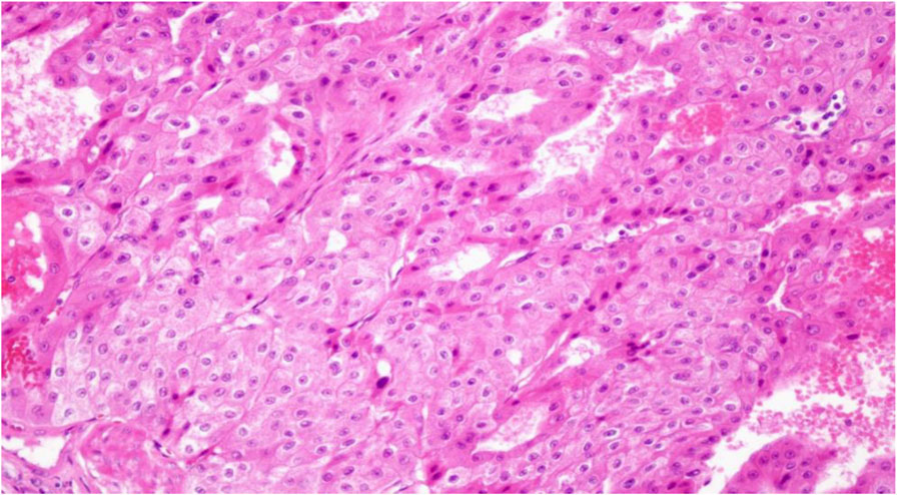
Case 2 with chromophobe cell carcinoma. HE×200

**Fig. 2:**
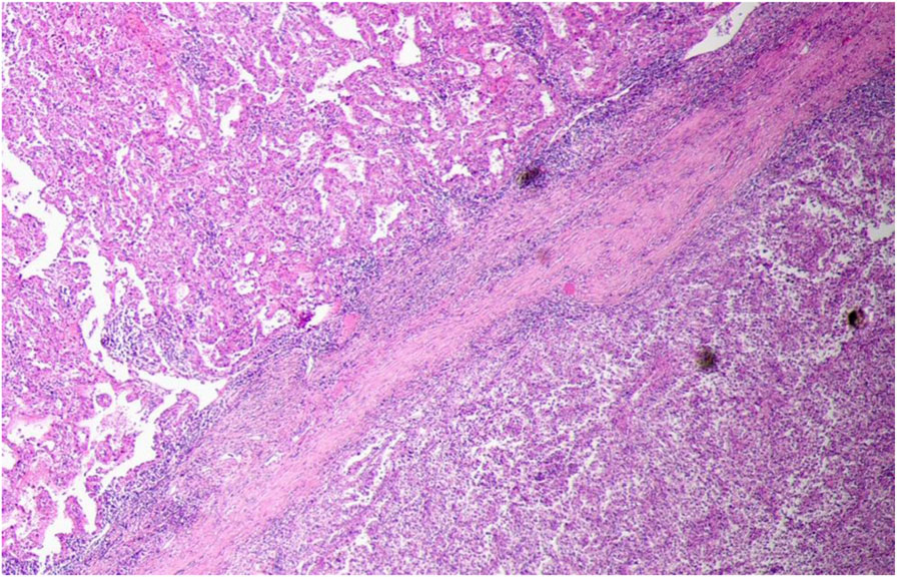
Case 8 with partially clear cell and partially papillary carcinoma. HE×40

The tumors’ median diameter was only 4.1 cm, due to early tumor stage and low degree of malignancy. Pathologic analysis showed that 17 (89.5%) patients had clear cell carcinoma (1 was cystic renal carcinoma) (Table 2), 1 had chromophobe cell carcinoma (Fig. 1), and 1 had partially clear cell and partially papillary carcinoma (Fig. 2).

Tumor grade classification showed that 1 patient had Fuhrman grade 1 disease, 3 had grades 1–2 disease, 5 had grade 2 disease, 2 had grades 2–3, and 4 had grade 3 (4 patients were not recorded). Fourteen patients (73.7%) were identified with stage pT1, and 9 of them were with stage pT1a. Only 2 patients were identified with locally advanced malignancy at stages pT3a and pT3b, respectively. The remaining 3 patients were identified with pT2 (Table 2).

### Treatments and outcomes

Operations were performed on each patient, 4 patients had partial nephrectomy, and 15 had radical nephrectomy (1 had removal of inferior vena cava cancerous suppository). Acknowledging the immunal deficiency, we recommended that all the patients with Furhman grades 2–3 or 3 accepted cytokine therapy, but only 2 patients with Furman grades 2–3 (histological type: clear cell carcinoma, partially clear cell, and partially papillary carcinoma) accepted cytokine therapy including IL-2 and IFN. During the median follow-up of 35 months (range from 14 months to 96 months), no patients occurred tumor recurrence. Two patients died of lung metastasis one year and six months after surgery respectively, even though 1 accepted full dose targeted therapy (sorafenib) for 3 months, and 1 refused adjuvant therapy. The remaining 17 patients are still alive at a median follow-up of 34 months; however, 1 patient lives with lung and brain metastases at the last follow-up of 35 years after surgery who underwent radical nephrectomy. This patient accepted sorafenib for 4 months, sunitinib for 6 months, and then axitinib for 3 months when diagnosed with lung metastasis 3 months after surgery, but he refused the immunal therapy (PD-1/PD-L1 inhibitors) considering the side effects and economic factors (Table 2).

## Discussion

We reviewed 19 cases diagnosed with RCC with HIV infection in Beijing Youan Hospital which is the largest HIV follow-up center in China. Our study is the largest published series about this kind of crew at present.

The average onset age of RCC in the general population is more than 60 with a male preponderance [10], while our study shows an earlier age of 51. Similarly, in an Australian statewide series of 7 HIV-positive patients, the median age of RCC diagnosis was also slightly younger at 52 years old [4]. Smoking seemed like a risk factor in A Transatlantic Case Series report, in which 7 of 9 patients had a smoking history [9]. However, we did not find conclusive evidence in our series, due to only 5 patients had a long smoking history. We cannot draw the conclusion that hypertension is the risk factor, because only 4 patients are complicated with hypertension, while smoking, obesity, hypertension, and chronic kidney disease had already been identified as risk factors in the general population [11]. The majority of cases presented as incidental findings screened out by imaging examination, while no patients presented typically triad syndrome (hematuria, flank pain, and abdominal mass), and only 1 patient presented hematuria and flank pain. This phenomenon was consistent with the general population because of the adhibition and diversity of screening methods [12]. In our study, we investigated patients’ immunal status in terms of CD4^+^ T lymphocyte cell count and HIV viral load, and we found no evidence on the relationship between immunal deficiency and tumor progression, even though the cases with no regular HAART treatment.

To our surprise, 73.7% of the patients had stage pT1 tumors in the case of immunal deficiency, which may predict better outcomes, and this proportion was close to that of the general population (75%) [13]. In our series, patients with patholigical classification of clear cell carcinoma accounting for 89.5% of all cases were slightly higher than the proportion of the general population (70–85%) [14]. Fuhrman grade is the most widely accepted histologic prognostic factor [15]. A large-sample study of 5453 patients in the USA showed that patients with Furhman grade 2 and 3 jointly accounted for 71.4%, and grades 1, 2, and 3 all had good outcomes [16]. In our report, 11 patients(73.3%) with Fuhrman grades 2 and 3 which was close to the above research also had good outcomes.

All the patients underwent operations successfully which were selected in accordance with general population, and 15 patients with radical nephrectomy and 4 with partial nephrectomy. The 4 patients who underwent partial nephrectomy are still alive without local recurrence or distant metastasis with mean follow-up of 43 months, the same outcome as in general patients [17]. There were a large number of literatures on radical versus partial nephrectomy for cT1 renal cell carcinoma. A recent study consisted of a total number of 2459 adults in the USA who were treated with radical or partial nephrectomy. It was documented that radical nephrectomy was associated with an increased risk of chronic kidney disease compared with partial nephrectomy, but there was no statistically significant difference in cancer-specific mortality (CSM) or all-cause mortality (ACM) among patients with cT1 RCC between radical nephrectomy and partial nephrectomy [18]. We cannot draw the same conclusions as the above study because of inherent defects of our study, but the 4 patients with partial nephrectomy did have good outcomes considering the immunal deficiency. More randomized controlled studies are necessary to confirm the good prognosis of partial nephrectomy versus radical nephrectomy in HIV-positive population, so as to reduce concerns that partial nephrectomy may predispose to recurrence and metastasis because of immunal deficiency special for the young HIV-positive population.

We have little experience to share on adjuvant treatment selection, due to good outcomes of our cases. We recommended patients with Furman grades 2–3 and grade 3 accepted cytokine therapy; however, only 2 patients agree to the scheme considering of side effects. We used targeted therapy in 2 patients with lung metastasis, but 1 patient still occurred brain metastasis and 1 died of lung metastasis. Checkpoint inhibitors have been identified to induce significant responses in RCC [19]. However, due to the role of PD-1^+^ T cells in HIV transcription in treated aviremic individuals and concerns of unforeseen side effects [20], the cases of cancers with HIV infection were rarely reported. In a recent literature, 16 HIV-positive nivolumab recipients were identified, including 8 non–small-cell lung cancer patients, 2 Hodgkin lymphoma patients, 2 RCC patients, and 4 off-label cancer patients. One of RCC patients was unable to assess side effects, response to treatment, and outcomes. The other RCC patient received 3 doses of Nivolumab with poor outcome and occurred grade 3 pneumonitis [21]. The latest study including 17 HIV-positive cancer patients showed that checkpoint inhibitors seemed like to have comparable efficacy and tolerable adverse effects, and CD4^+^ T lymphocyte cell count and viral load were not affected. In this study, only one RCC patient with stage III responded to immunal therapy with stable disease and had slight side effects [22].

## Conclusions

In our study, RCC Patients with HIV-positive were similar to the general population in terms of clinical characters, treatment measures, and pathology. RCC patients with HIV positive seemed like to obey the same clinical practice guideline as in the general population. To our delight, the outcomes of patients with partial nephrectomy are not inferior to patients with radical nephrectomy. Young HIV-positive patients with early-stage tumors would benefit so much from the nephron-sparing surgery, and it is no need to worry about the local recurrence and distant metastasis caused by immunal deficiency. Further study are still warranted to investigate RCC features in HIV-positive population. Checkpoint inhibitors are revolutionizing cancer therapy; however, more experience still needs to be learned in HIV-positive population.

## Data Availability

All data generated or analyzed during this study are included in this published article.
